# Identifying disrespect and abuse in organisational culture: a study of two hospitals in Mumbai, India

**DOI:** 10.1080/09688080.2018.1502021

**Published:** 2018-08-13

**Authors:** Neha Madhiwalla, Rakhi Ghoshal, Padmaja Mavani, Nobhojit Roy

**Affiliations:** a Co-ordinator, Centre for Studies in Ethics and Rights, Anusandhan Trust, Mumbai, India; b Senior Programme Officer, Centre for Studies in Ethics and Rights, Anusandhan Trust, Mumbai, India; c Associate Professor, Department of Obstetrics and Gynaecology, Seth G.S. Medical College and K.E.M. Hospital, Mumbai, India; d Head, WHO Collaborating Centre for Research on Surgical Care Delivery in LMICS, Surgical Unit, BARC Hospital, Mumbai, India

**Keywords:** childbirth in LMICs, disrespect and abuse, obstetric practice in LMICs, provider behaviour, hospital culture

## Abstract

This paper draws on findings from a qualitative study of two government hospitals in Mumbai, India, which aimed to provide a better understanding of the institutional drivers of disrespect and abuse (D&A) in childbirth. The paper describes the structural context, in which government hospital providers can exercise considerable power over patients, yet may be themselves vulnerable to violence and external influence. Decisions that affect care are made by a bureaucracy, which does not perceive problems with the same intensity as providers who are directly attending to patients. Within this context, while contrasting organisational cultures had evolved at the two hospitals, both were characterised by social/professional inequality and hierarchical functioning, and marginalising women. This context generates invisible pressures on subordinate staff, and creates interpersonal conflicts and ambiguity in the division of roles and responsibilities that manifest in individual actions of D&A. Services are organised around the internal logic of the institution, rather than being centred on women. This results in conditions that violate women's privacy, and disregards their choice and consent. The structural environment of resource constraints, poor management and bureaucratic decision-making leads to precarious situations, endangering women’s safety. With the institution's functioning based on hierarchies and authority, rather than adherence to universal standards or established protocols, irrational, harmful practices endorsed by senior staff are institutionalised and reproduced. A deeper focus on organisational culture, embedded in the discourse of D&A, would help to evolve effective strategies to address D&A as systemic problems.

## Résumé

Cet article est fondé sur les conclusions d’une étude qualitative de deux hôpitaux publics à Mumbai, Inde, avec pour but de mieux comprendre les facteurs institutionnels du manque de respect et de la maltraitance à l’accouchement. L’article décrit le contexte structurel, dans lequel les prestataires de l’hôpital public peuvent exercer un pouvoir considérable sur les patients, mais peuvent être eux-mêmes vulnérables à la violence et aux influences externes. Les décisions qui agissent sur les soins sont prises par des bureaucrates, qui ne comprennent pas les problèmes avec la même intensité que les prestataires qui traitent directement les patients. Dans ce contexte, alors que les cultures organisationnelles contrastées avaient évolué dans les deux hôpitaux, les deux étaient caractérisées par des inégalités sociales/professionnelles et un fonctionnement hiérarchique, et par la marginalisation des femmes. Cette situation génère des pressions invisibles sur le personnel subordonné et crée des conflits interpersonnels et des ambiguïtés dans la division des rôles et des responsabilités qui se manifestent dans des actes individuels d’irrespect et de maltraitance. Les services sont organisés autour de la logique interne de l’institution, au lieu d’être centrés sur les femmes. Cela aboutit à des conditions qui violent l’intimité des femmes, et méprisent leur choix et leur consentement. L’environnement structurel de limitation de ressources, de piètre gestion et de prise de décision bureaucratique cause des situations précaires, mettant en danger la sécurité des femmes. Avec un fonctionnement de l’institution fondé sur les hiérarchies et l’autorité, plutôt que sur le respect des normes universelles ou des protocoles établis, des pratiques nuisibles et irrationnelles, approuvées par le personnel de supervision, sont institutionnalisées et reproduites. Une priorité accrue à la culture organisationnelle, ancrée dans un discours de mépris et d’abus, aiderait à faire évoluer des stratégies efficaces pour s’attaquer au manque de respect et à la maltraitance comme problèmes systémiques.

## Resumen

Este artículo se basa en los hallazgos de un estudio cualitativo de dos hospitales gubernamentales en Mumbai, India, cuyo objetivo fue mejorar la comprensión de los impulsores institucionales de la falta de respeto y el maltrato durante el parto. El artículo describe el contexto estructural en que los prestadores de servicios de salud en hospitales gubernamentales pueden ejercer considerable poder sobre las pacientes y a la vez ser vulnerables a violencia e influencia externa. Las decisiones que afectan los servicios de salud son tomadas por una burocracia, que no percibe los problemas con la misma intensidad que los prestadores de servicios que atienden directamente a las pacientes. En este contexto, aunque en los dos hospitales evolucionaron culturas institucionales contrastantes, ambas estaban caracterizadas por desigualdad social/profesional, por funcionamiento jerárquico y por marginar a las mujeres. Este contexto genera presiones invisibles en el personal subordinado y crea conflictos interpersonales y ambigüedad en la división de funciones y responsabilidades, que se manifiestan en acciones individuales de falta de respeto y maltrato. Los servicios están organizados en torno a la lógica interna de la institución, en vez de centrarse en las mujeres. Esto produce condiciones que violan la privacidad de las mujeres y hacen caso omiso de sus decisiones y consentimiento. El ambiente estructural de limitaciones de recursos, administración deficiente y toma de decisiones burocráticas ocasiona situaciones precarias, que ponen en peligro la seguridad de las mujeres. Con el funcionamiento de la institución basado en jerarquías y autoridad, y no en el cumplimiento de normas universales o protocolos establecidos, se institucionalizan y reproducen prácticas dañinas e irracionales aprobadas por el personal de nivel superior. Un enfoque más profundo en la cultura institucional, arraigado en el discurso sobre la falta de respeto y el maltrato, ayudaría a formular estrategias eficaces para tratar la falta de respeto y el maltrato como problemas sistémicos.

## Introduction

Disrespect and abuse (D&A) of women during childbirth need to be understood not as random acts or aberrant behaviour of individual providers, but as systemic problems.^1^ Various facility factors, such as overcrowding, shortages, hospital rules and policies have been implicated for accentuating D&A.^2,3^ In highly stratified societies, typically, very hierarchical and undemocratic relationships form between providers and women.^4,5^ Less educated women, rural women, women with a stigmatising condition like HIV, unmarried mothers, women seeking care in government hospitals have been documented to face greater levels of (D&A).^6–8^

Presently, the discourse on D&A accommodates providers’ perspectives to the extent that they provide explanations for the providers' behaviour as reported by women. Few studies have attempted to locate particular abusive, irrational, harmful practices in a specific hospital environment, with its rules, relationships and power structures.^9^ For example, in Brazil, routine practice of episiotomy was embedded in a stratified health system where poor women were relegated to limited and under-resourced government institutions in which they became unwitting subjects for clinical training.^10^ Various harmful and painful interventions to hasten labour (manual dilatation of the cervix and routine induction) were part of a strategy to “clear” the wards in order to cope with the high patient load in Mexico.^11^ Nurses’ behaviour towards poor women was embedded in the legacy of a colonial health system characterised by deeply unequal and almost coercive relationships between patients and providers in South Africa.^12^ A review of research from sub-Saharan Africa concluded that individual providers’ attempts to exert power and control and maintain their higher status vis-a-vis labouring women were explained by an “institution-centred, medicalised and hierarchical” model of maternity care.^13^

These studies explored aspects of organisational dynamics to the extent that these produced particular practices. However, understanding the organisational context more fully would help explain how and why patterns of behaviours and actions emerge and how they get reproduced within the institution. Midwives perceived unsupportive working environment, unsatisfactory interactions with women, lack of training and the neglect of best practices as barriers to providing perinatal care.^14^ Burnout and moral distress result from the interaction of social factors such as the low status of midwifery, the low wages of health workers, and organisational problems such as poor skill development and training and inter-professional conflicts.^15^ Working in very constrained conditions with highly disadvantaged women engenders a fatalism about patient deaths among frontline workers^16^ Official reviews inevitably end in attributing blame to lower level functionaries, further demoralising them.^17^ Inevitably, these result in an organisational culture where women are devalued and D&A is normalised.

In this paper, we examine the organisational culture of two government hospitals, as reflected in providers’ articulation of their beliefs, their descriptions of work and their analysis of daily situations, in order to understand the drivers of D&A in institutional obstetric care. Our analysis of provider narratives is supplemented by observation and review of documents.

Our study, focusing entirely on professional providers (doctors and staff nurses), was conducted prior to the discourse on D&A gaining currency. However, our findings still resonate with the feminist discourse on the violation of women’s rights in the health system that echoes most, if not all, of the contemporary concerns. Studies which had documented women’s experiences of medicalised childbirth in the Indian context had pointed to a range of problematic areas. This included the ridiculing of women’s cultural beliefs and talking down to them,^18^ the common use of physical force and violence,^19^ coercion to accept birth-control,^20^ asking for husband’s consent for abortion,^21^ devaluation and penalisation of women’s experience of pain,^22^ discrimination based on caste,^5^ and a deep-rooted gender bias that produced apathy and neglect.^17^

We also draw on parallel literature that criticises the over-medicalisation of childbirth and the ethics in contemporary clinical practice. This literature identifies problematic areas including the overuse of interventions such as episiotomies^10^ and Caesarean sections.^23^

Thus, in this study, we focus on domains of practice where violations or ethical problems had been commonly observed, namely, provider-patient interactions, cultural and social issues encountered in care-giving, management of labour pain, routine practices and procedures associated with normal vaginal deliveries, management of complications and post-delivery contraception.

## Methods

The study was done in two government hospitals in the metropolitan Indian city of Mumbai between 2010 and 2013. We studied obstetric practice in one tertiary care teaching hospital attached to a government medical college (MC) and a government secondary care hospital located in an extended suburban district of the Mumbai urban agglomeration (SH). Both of these hospitals were representative of their sector in terms of organisational structure, workload and resource availability.

Two of the co-investigators (NR and PM) are practicing clinicians, the latter being an obstetrician. One of the researchers (NM) had extensive experience of government hospitals due to her prior research experience. This multi-disciplinary team had numerous discussions and debates to evolve a conceptual framework that would reflect their diverse perspectives, even as it spoke to the different disciplines that they represented – social sciences, bioethics and medicine.

Based on these inputs, we refined the specific research question, demarcated domains of inquiry and designed data collection tools. We organised consultation processes prior to the conduct of the study. One involved international and Indian researchers and academics/researchers who undertook scientific review of the project. The other involved practising professionals from government and private hospitals in Mumbai and focused on the use of language, the approach to fieldwork, probable logistical and ethical problems that we could expect to encounter.

The study used qualitative methods, including in-depth interviews, key informant interviews and observation. Fieldwork was carried out by a team of trained social science researchers (NM, RG and three others). All the researchers were fluent in English and one or both of the dominant local languages, Hindi and Marathi. All the researchers were women, of Indian origin and, barring one, resided in Mumbai.

As a first step, we compiled information about the institutions using an observation checklist and information from key informants to draw up an institutional profile. We compiled statistics on the institutions’ performance, including the numbers of deliveries conducted, C-Section rates and patient outcomes. We made note of the physical infrastructure, layout and design of the space, provisions for allied services such as pathology, radiology, pharmacy and the condition of these facilities. We also documented stated policies regarding recruitment, supervision and monitoring of staff and the challenges faced by the institution in this regard. We documented norms related to the division of labour between various categories of staff and decision-making related to patients. This profile was very useful for understanding the formal framework within which the organisation functioned. It also helped the team to identify areas where actual practice was in contradiction with stated norms.

Simultaneously, the researchers who would be involved in conducting interviews and observations had extensive informal interactions with experienced obstetricians, nurses and hospital administrators who had experience of working in similar settings, to gain an understanding of frequently used technical terms and abbreviations as well as the discursive language that marked that sub-culture, embedded in slang, jokes, “open-secrets”, dictums and stock phrases. This was followed by a period of 2–3 weeks spent in the hospitals interacting with staff and observing everyday routines. Apart from gaining familiarity with the hospital’s functioning, this served as an opportunity to offer clarifications and explanations to staff who were curious about our presence and, to the extent possible, reduce the possibility of our presence altering the environment.

In the government hospitals, we had access to clinics, waiting areas, staff rooms, nursing stations and antenatal and postnatal wards. These were areas accessible to the female relatives of the childbearing women. Additionally, we had been allowed to visit the labour room and operation theatre when it was not in use. As it was focused on providers, we did not study women or their relatives. We specifically avoided being present in situations or locations where we would invade the privacy of patients. Our interactions with women and their relatives were limited to responding to their queries. Some casual conversations, as which typically occur among bystanders, took place often. However, it was explicitly known to the hospital management and staff that we would not be interviewing patients.

All professional providers connected with obstetric services (obstetricians, residents, nurses, social workers and hospital administrators) in the two government hospitals were included in the sampling frame. Overall, we interviewed 16 presently employed obstetricians (residents and consultants), 5 formerly employed obstetricians, 7 nurses and 2 hospital administrators connected to the obstetrics services across these two hospitals. Overall, we interviewed about half of obstetricians present and about one-fourth of the nurses. Written consent was obtained from each participant. A formal letter of permission signed by the Dean/Hospital Superintendent was available and a copy was shared with each participant. However, most participants had already received information about the study from the hospital authorities, conveyed via their supervisors.

Interviews were conducted over 1–3 sessions, mostly in the wards. Each session was interrupted by the participants to attend to patients or finish paperwork. If the researchers perceived that the participant was too busy, despite having consented to be interviewed, they proactively sought an appointment on another day and or volunteered to wait. Almost all the interviews were audio-taped. We conducted their interviews in the language of the participants’ choice. We shared the interview guide with each participant at the beginning of the interview. This included questions on their personal background, motivations for joining their chosen profession, training, routine practices related to conducting normal deliveries, pain-relief, post-partum contraception, complicated cases, referral and transfer, and clinical audit. We also asked them to describe the women who sought care and elicited accounts about individual women or incidents that they found challenging and rewarding.

While we waited to meet a participant, which was a period of 2–5 hours, we observed provider-patient interactions, conversations and exchanges between staff members, routines of individual providers as well as unusual events. Typically, we were present in the hospitals between 10 am and 8 pm. We made notes and included specific probes/questions in the interviews with participants related to these. These questions typically related to underlying conflicts among staff, undocumented challenges in providing care, stress and workload, adaptations to cope with specific problems, actions which aimed at building solidarity and co-operation between staff.

Interview transcripts and field notes were coded and analysed using WeftQDA. Two stage coding was undertaken. Minute coding was done to identify specific areas of discussion, e.g. patient profile, discharge procedures, pain relief. These were combined into themes – e.g. provider attitudes, resource management, grievance redress and supervision.

The study was approved by the Multi-Institutional Ethics Committee to which the Anusandhan Trust was affiliated. It was also approved by the ethics committee of MC. We obtained official permission to conduct fieldwork in SH, which did not have any ethics committee.

## Results

### The broader context

As a result of high workload and adverse working conditions, delivering obstetric care was viewed as problematic in these hospitals. The participants recounted problems related to poor management, socio-cultural barriers in delivering care to patients, shortages and misallocation of resources. There was an acknowledgment that mistreatment of women in the form of shouting and physical coercion existed, although this was not necessarily perceived as abuse. Several other aspects of practice, such as use of pain relief medication, episiotomies, induction of labour and post-partum contraception, were perceived to be unproblematic.

Both hospitals offered free services and usually did not refuse admission to any woman. However, the provision of services was not located in a framework of civic rights. Despite a policy shift away from coercion, we found women were being compelled to accept birth control after delivery. There was an informal code in both hospitals that women must accept tubal ligation after two deliveries and IUD insertion after the first. The typical strategies used to pressurise women were refusing discharge, threatening not to conduct the procedure or banning her from the hospital. Typically, consent for these predetermined choices was negotiated when women were at their most vulnerable.

“What we prefer over here; what I have been doing here is; I am telling my juniors and have been told by my seniors; is that if the lady is in her active phase of labour, it is the best time to talk to her about TL [tubal ligation] … They are very receptive at that time and they are exclusively with me at that time inside the labour ward … They understand what pain it is, how it is good to not have it once again.” *(KSDE, Senior resident, MC, Female)*

There was no entitlement to a specific set of services and resources. The women and their relatives were largely expected to fill gaps. A study conducted in a similar context has documented women and their relatives being compelled to clean the wards and wash their own clothes.^24^ While we did not observe these practices, relatives were asked to take on many tasks officially to be performed by orderlies. In both hospitals, obstetric services were spread across different floors. Laboratory and radiology services were located in different wings. Thus, relatives were required to push the gurney, transport samples, bring medicines, etc. Not only did this impose economic burdens on the family, it led to overcrowding and chaotic conditions in the wards.

Staff typically attributed these problems to the municipal corporation, which controlled vital aspects of the hospital functioning such as admission policies, charges, staffing, documentation and some aspects of clinical work. This displaced decision-making from the domain of professional to bureaucratic practice. Hospital managers either had to resort to informal means to overcome challenges or cope with existing resources.

“You have reason to get a little frustrated, agree. But you also have ways of getting it done. You have to literally explain about the requirements, and you have to give reasoning, perfect reasoning for that. [You need] a superior who is backing you in such types of job. He should be like a godfather.” *(VPJ, Senior Manager, SH, male)*

“I cannot say we do not have enough nurses because the corporation does not have sanction for appointing more people. We have to do with whatever we have.” *(KSVB, Senior Faculty, MC, male)*

The price of seeking patronage was accommodation of local elected representatives’ demand for special services or attention to particular patients, who were popularly called “note-cases”, as they brought hand-written notes of recommendation. In both hospitals, there were expressions of disappointment about the top management’s inability to protect the frontline staff from feeling pressurised.

“I was attending a high risk woman and another woman who was not due for another 4 to 5 hours came in and along with some local party workers, demanding that I attend to her immediately. I refused, so they threatened me and went out and soon there were more people. They called up the superintendent and complained against me. I explained, but I was compelled to attend to the other woman. They [hospital management] told me that “nothing could be done.” They should have stood by me … but then, maybe had I been in their position I too would have done the same.” *(VAN, Senior Resident, SH, male)*

### Specific organisational contexts and cultures in the hospitals

Within this paradoxical context of arbitrary power over women and subordination to the bureaucracy, the hospitals had evolved quite differently in response to their specific contexts. SH was located at the periphery of the city. Typically, most women here were “booked patients”, i.e. registered here during pregnancy and also delivered here. As the secondary care referral hospital (SH) received women from a defined catchment area, there was relatively more familiarity with women. Additionally, although it had similar provider:patient ratios to the medical college hospital (MC), the predominance of uneventful births created a more relaxed working environment.

Typical of peripheral centres, hospital management had little effective control over the specialists on the staff; there was high turnover, absenteeism and covert engagement in private practice. By administrative arrangement, a nearby private medical college placed residents here for clinical training, who became the mainstay of medical services. At any given time and particularly from late afternoon till morning, residents were the only medical staff present.

Private anaesthetists had been empanelled to provide services. Departments such as radiology and pathology did not function round the clock. This inevitably left residents in charge of making decisions, not merely based on their evaluation of the woman’s medical condition, but taking into account various external constraints. There were no protocols for the management of emergencies or routine cases. This environment led them to make ad hoc decisions. Hence, sudden transfers to fairly distant tertiary care hospitals were not infrequent.

“If there is an emergency, like the baby [foetus] is in distress, we have to take in the patient even after [our shift]. But sometimes the anaesthetist is not available, or refuses to come, then we [residents] only have to hand over the patient to the other hospitals.” *(VAV, Senior Resident, SH, female)*

There were no organisational safeguards for dealing with sudden and life-threatening emergencies which were beyond the competence of residents or where there was not enough time to transfer the patient. Such incidents, when they occurred, exposed the precarious conditions of care at the hospital.

“Inverted uterus is a life threatening situation. We shifted the woman from the labor ward to the main OT. Fortunately the anaesthetist was there. We induced and immediately delivered the baby. Within 2 minutes we repositioned the uterus. Had I been not there in that shift the patient would have been dead since no one knew how to reposition the uterus.” *(VAM, Head of Ob/Gyn, SH, male)*

However, as emergencies were not frequent events, there was no attempt to discipline the medical staff more stringently. Instead, the hospital administration relied on its nursing staff to keep the hospital functional. The nursing staff were a relatively cohesive and stable group. They had a complex and ambivalent relationship with the residents. They were usually much older and skilled at performing clinical tasks. At the same time, the residents had greater clinical knowledge and could conduct surgeries. Residents were also officially responsible for conducting vaginal deliveries, although these are often conducted by nurses. This often resulted in a conflict at the frontline about the division of work and responsibility.

“Some doctors will say that he will have his lunch and only then come in for the delivery and if the patient is in 2nd stage then we will deliver her. We can’t delay the delivery and put the patient at risk!” *(VLD, Staff Nurse, SH, female)*

Also, nurses were still seen as doing less skilled and “dirty work”, a phenomenon which has been documented.^25^ This undermined the importance of their inputs. To illustrate, residents left it to nurses to “prepare” women for internal examination, which actually subsumed a process of getting informed consent and protecting their dignity.

“When the patient comes in the examination room, generally they are wearing “internal clothes”. So telling them to remove it is really embarrassing. Of course, nurses are there [and] they will be explaining them these things … So what we tend to do is go outside and wait for the patient to get [into] position and we just do the internal examination.” *(VAN, Senior Resident, SH, male)*

However, nurses determined the working environment. Thus, wards were managed more informally. Nurses engaged in more informal interactions with women and their relatives. Thus, by and large, even medical staff at SH had much more insight into their patients’ lives and described positive interactions with them.

“She [a woman] was not getting proper sleep and maybe she was not having proper emotional support at that time from the husband and the family, so she was very much frustrated and, in addition, that patient had some psychiatric problem so she … absconded.” *(VRB, Senior Resident, SH, male)*

“A few days back, a patient of mine, she got me 2 kilos of fish! I had done her LSCS [Lower Segment Caesarean Section] and had discharged her long back.” *(VAN, Senior Resident, SH, male)*

We also observed another woman who had “absconded” following her previous delivery and was pregnant again. The nurses recognised her and began to reprimand her. The woman, on her part, made attempts to cajole and humour them and she was eventually admitted.

However, while they were more informal, the nurses’ treatment of women was not necessarily less arbitrary or aggressive. Shouting and the use of physical force on women during labour was openly acknowledged by the participants. As their intention was to protect women and babies, they perceived the overt violence as justified.

“Suppose the head has come out and she needs to bear down, but she is not pushing. Then we have to shout, because if she doesn’t push, both will suffer. Both might die. Now people say later,‘that doctor shouted, sister shouted, sister slapped’. Now, I know these things are not right, it is against human rights, [but] they do it for the good of the baby.” *(Matron, SH, female)*

The dominance of nurses, a more homogenous and familiar population of patients and a preponderance of uneventful deliveries created an environment in which women’s individuality was somewhat preserved. However, the inter-professional conflict, an absence of formal frameworks for regulating clinical practice and ineffectual management created a stressful environment for the staff and posed risks for women.

Being a teaching hospital, MC had an abundance of medical staff and a greater focus on academics and research. It was also much more generously staffed and equipped. Apart from its “booked patients”, this hospital received women who were referred or transferred from smaller government hospitals across the city and its periphery. MC received a much higher proportion of women experiencing complications. Several maternal deaths also took place here, mostly among transferred women. Following such events, the staff of the obstetrics department were called in for an audit, which involved the hospital staff as well as the health department bureaucracy. As a result, although a majority of women had normal vaginal births, there was a heightened focus on risk. Complementarily “saving lives” became the overarching ethic for the hospital.

“Whatever we do, we have to do to save the patient … A person who is very critical will die. [Still] I do not think anybody would have been saved outside this institute only because they had more money, or better equipment. No way!” *(KSVB, Faculty, MC, male)*

An acute awareness of “risk” and a preoccupation with “saving lives” did translate into an importance placed on protocols. It is interesting that participants did not make any reference to international or national treatment guidelines or protocols. They referred largely to internal rules and a certain managerial process which was focused on preventing decision-making by untrained residents.

“After 4 p.m., if a critical patient comes I will see the patient and I will inform them [senior team members], they will come and see, and whatever decision is given by them I will inform to the boss [Head of the Unit] by phone [sic]. Then we describe the case, whatever investigations are available, like … this is the findings of a qualified person. The lecturer advises what is to be done. Then the HOU [Head of the Unit] may modify the decision or they may say OK you may go ahead with the same decision.” *(KsDe, Senior Resident, MC, female)*

**Table 1.** Details of the study sites and respondents

	Respondents			
Institution	Residents	Specialists	Nurses	Others
Tertiary care hospital attached to a medical college (MC). 240 obstetric beds out of 3000 in the hospital. 7000 deliveries conducted annually. Ob/gyn department with six units	4 final year residents (senior residents) pursuing MD (female)	2 professors (1 male, 1 female), 2 associate professors (female), 1 lecturer (male)	1 matron, 1 sister-in-charge, 1 staff nurse (all female)	5 alumni (2 female and three male) practicing in a non-profit medical college as faculty
Secondary care referral hospital (SH). 70 obstetric beds out of 300 in the hospital. 3500 deliveries conducted annually. Ob/gyn department with three units	3 senior residents pursuing DGO (two male, one female)	3 senior consultants (male), 1 junior consultant (female)	1 matron, 1 sister-in-charge (obstetric ward), 1 sister-in-charge (NICU), 1 staff nurse(obstetric ward) All female	1 medical superintendent (male), 1 resident medical officer (male)

Complementing this system of decision-making was a supervision system based on attributing blame. Repeatedly residents referred to anxiety about being wrong. In turn, they referred to correcting mistakes of their juniors, rather than mentoring. Medical staff did not collaborate with nurses, who were almost excluded from medical work and relegated to record-keeping. In turn, their supervision of hospital orderlies was also based on directives and reprimands.

“The class IV staff [hospital orderlies] is overworked – at times one man will have to do the work of three men. And then when everybody will shout at him for being late, how much can the poor fellow bear? And you know what, working in this place, I have got so used to shouting while talking, that even at home, I shout when talking!” *(Assistant Matron, MC, female)*

The focus on “saving lives” also resulted in local adaptations aimed at providing better care to selected women, whose needs were more acknowledged than usual. A system of shifts and rotations was instituted to ensure that these women who were classified as “high-risk” were treated by the same unit whenever they arrived at the hospital. While the residents unequivocally complained about long working hours and heavy workloads, they were all appreciative about this system.

“They know the patients from the beginning … the patient may not have the papers when emergency occurs. But the registrar knows that so and so is the high risk patient of our unit. So that is the basic idea behind this thing – that patients are not mismanaged. He still knows that this is high risk due to this reasons.” *(KSDH, Senior Resident, MH, female)*

In contrast, the general population of women seeking obstetric care would rarely interact with the same provider more than once or twice. Thus, provider-women relationships here were considerably more formal. We never heard women referred to by their name in this hospital, unlike SH. While narrating incidents, residents and nurses who had the most contact with women always described them by their medical conditions, rather than their social or family background. While a bias against certain communities, such as Muslims, was pervasive, it became more pronounced in this impersonal environment.

“My severe heart disease patient, a Muslim woman, whom we counselled and sent home after doing a MTP, telling her to not get pregnant again, she comes back with pregnancy within one month not even two, three months. Sometimes you just lose it.” *(KRP, Senior Resident, MC, female)*

In both hospitals, administering pain relief was not part of the routine process. In general, staff did not perceive any responsibility to respond medically to women’s expressions of pain. While they differed in their perception about women’s experience and ability to tolerate pain, participants in this hospital were more likely to judge women.

“See, pain is a very subjective phenomenon; and also depends upon the sensitivity of the woman, her ability to tolerate pain. Probably women who are more pampered by their husbands will not be able to bear pain.” *(KAG, Senior Resident, MC, female)*

The distance from women, lack of insight into their personal lives and a preoccupation with safety combined to obscure violation of women’s rights. The unwritten protocols which were transmitted through the hierarchy also overwhelmed the space where reflection and discussion was required. An ethical dilemma, which required the woman’s choice to also be evaluated and respected, was also rendered unproblematic and resolved mechanically.

“We then think of the mother as well as the baby. We cannot take up a patient with a haemoglobin level of 5 for C-section. There might not be time for us to wait and give her 3 or 4 bottles of blood and then do a C-section. We face a lot of dilemma these times. But we prefer having healthy mother, she can have babies later.” *(KRH, Senior Resident, MC, Female)*

An emphasis on formal structures and hierarchies was much more pronounced in the organisational culture on MC. However, rather than relying on established guidelines, they were more hierarchical, based on following directives and deferring to seniors. In the absence of supportive supervision, this system produced an environment of conflict and mistrust. An attempt was made to facilitate the care of women who faced medical risks, but staff did not, generally, perceive women’s personhood.

## Discussion

Using existing frameworks,^7,26^ we examine the implication of organisational culture for the D&A of women. We identify drivers and their relationship to three key domains of problematic practice.

(1) *Behaviours aimed at obtaining women’s compliance to staff’s directives - use of physical force, threats, coercion, detention etc*.

As noted above, the hospitals were located in a complex structural context. On the one hand, very unequal social relationships between women and providers led to a certain “othering” of women, due to which providers felt affectively distanced and tended to blame women. In addition, exposure to formal and informal actions by external agents made them see, in general, all women and their relatives as a threat, for which they sought police protection and administrative safeguards. The consequence of both these factors was poorer rapport and less engagement with women. A paternalistic belief in their entitlement, even responsibility, to adjudicate women’s best interest made overtly coercive and violent actions justifiable and morally acceptable.

However, within this largely paternalistic framework, patterns of behaviour differed. In SH, the nurses’ dominant role allowed women to emerge as persons, who though subordinate could attempt to negotiate and shape relationships. Staff also experienced positive non-clinical encounters with women. Arguably, such encounters could challenge prejudice and encourage providers to view engagement with women as a part of their professional role. However, as inter-professional training was unheard of, these encounters did not gain any lasting significance.

(2) Behaviours and practices that displaced women from the focus of care – non-consented care, violation of privacy and confidentiality, being left alone, not being supported, etc.

It was evident, in both hospitals, that women were not the focal point for the design or management of services. The design of the space, the organisation of services and distribution of tasks evolved from the internal logic of the institution. Thus, male relatives required to undertake heavy work and purchases in lieu of absent orderlies crowded the hospital wards, leading to invasion of women’s privacy, even while the design of the labour room made it impossible for women to have a lay companion during labour. Women could be left alone, even in the presence of skilled staff, if there was ambiguity about who was responsible for their care. Decision-making rarely involved women, but was guided either by exigencies, as in the case of SH, or by informal rules and protocols, as in MC. Women would experience these as sudden changes in treatment plans, arbitrary transfer or neglect. Contraception and abortion were offered by staff based on arbitrary rules and force was exerted to obtain compliance. The irrelevance of women to the processes of caregiving was so institutionalised that it did not even occur to staff to elicit their views. Moreover, the pressure to follow directives or to cope with inadequate conditions of care overwhelmed the space for women’s expression of choice. Women faced frontline providers, who appeared arbitrary and neglectful, but were responding to invisible pressures, which they had little power to resist.

(3) Acts of omission and commission that endangered women’s lives or made care-seeking more challenging – routinisation of procedures that were either harmful or not helpful, ad hoc treatment, referrals or transfers, etc.

Staff in both hospitals worked in imperfect environments, which were the result of poor management, administrative lapses and an out-dated mode of clinical practice. Frontline providers felt helpless to change the resource environment and management functioning. So they resorted to adaptation to ensure that women’s lives were not lost. This included improving care for selected women by enhancing their own workload or taking ad hoc decisions to transfer women, who could, technically, be treated at the hospital. Wherever they could, they extracted resources and labour from women and their families to cover the gap in provisioning. The locus of power rested in the corporation, which did not, so to say, have to face patients every day. Administrative rules and local power dynamics dictated the flow of funds, resources and personnel, rather than a proactive attempt to alleviate the suffering of patients and improve care for them.

Evidence-based practice was conspicuous by its absence, and, thus, out-dated, potentially harmful or unnecessary practices, if they were endorsed by influential seniors, were institutionalised. The relative unimportance of updating knowledge and aligning their practice to established standards meant that residents, who were the mainstay of care, could not refer to established protocols, but had to comply with their seniors’ directives. In the domain where directives were not available, they had to act intuitively within the boundaries of their competence. Even so, they remained vulnerable to blame and punitive action.

## Conclusion

This study was premised on the fact that not only do violations of women’s rights during maternity occur, but that providers openly acknowledge them and have explanations to offer. We explored the everyday working environment to uncover the systematic structures and processes that determine interactions between providers and women. Literature from the domain of quality of care and health systems studies has contributed to our understanding of the effect of underlying structures and processes on what happens at the point of care.^15^ The discourse on D&A has helped to centre the discussion on women, rather than viewing their experience of care as a marginal aspect of quality. Going forward, addressing D&A beyond the penalisation of individuals, will require a deeper engagement with institutions and health systems to understand how social inequalities, institutional structures and processes and individual agents interact to create an organisational culture, which produces violations and is in need of change.
